# Isolation and Characterisation of Culturable Thermophilic Cyanobacteria from Perak Hot Springs and their Plant Growth Promoting Properties Effects on Rice Seedlings (*Oryza sativa* L.)

**DOI:** 10.21315/tlsr2023.34.3.1

**Published:** 2023-09-30

**Authors:** Clement Kiing Fook Wong, Tzu Yee Chong, Ji Tan, Wey Lim Wong

**Affiliations:** 1 Department of Agricultural and Food Science, Faculty of Science, Universiti Tunku Abdul Rahman, Jalan Universiti, Bandar Barat, 31900 Kampar, Perak, Malaysia; 2 Department of Biological Sciences, Faculty of Science, Universiti Tunku Abdul Rahman, Jalan Universiti, Bandar Barat, 31900 Kampar, Perak, Malaysia; 3 Centre for Agriculture and Food Research, Universiti Tunku Abdul Rahman, Jalan Universiti, Bandar Barat, 31900, Kampar Perak, Malaysia

**Keywords:** Biofertilizer, Cyanobacteria, Hot Springs, Plant Growth Promoting, Thermophilic, Baja Bio, Kolam Air Panas, Penggalak Tumbesaran Pokok, Sianobakteria, Termofilik

## Abstract

Malaysia is home to a number of hot springs that are rich in microbial diversity including the photosynthetic cyanobacteria. Although this microbial community has been characterised based on metagenomics approach, the culturable thermophilic isolates have not been isolated and characterised extensively. Compared to the mesophiles, information on plant growth promoting (PGP) properties of these thermophiles remain largely untapped. As the amount of arable land for microbial bioprospecting is decreasing due to extensive human activities, the search for alternative source for microbial strains with PGP properties is important for the development of potential biofertilisers. This study sought to isolate and characterise culturable cyanobacteria strains from two local hot springs – Sungai Klah (SK) and Lubuk Timah (LT) located in Perak using morphological and molecular methods. The IAA production from the axenic cultures were measured. The PGP properties were also measured by priming the rice seeds with cyanobacterial water extracts. A total of six strains were isolated from both hot springs. Strains LTM and LTW from LT were identified as Leptolyngbya sp. whereas strains SEM, SEH, STH and STM were identified as *Thermosynechococcus elongatus*. All six strains produced IAA ranged from 670.10 pg/μL to 2010 pg/μL. The water extracts were found to increase the seed amylase activity of the rice seeds from 5th day of germination (DAG) to 10th DAG. In general, the IAA production and increased seed amylase activity might have contributed in enhancing the longest root length, shoot length and root-to-shoot (RS) ratio. To conclude, the thermophilic cyanobacteria from hot springs can be further exploited as a novel source of PGP microbes for the development of biofertilsers.

## Abstrak

Malaysia mempunyai beberapa kolam air panas yang kaya dengan kepelbagaian mikrob termasuk sianobacteria fotosintetik. Walaupun komuniti sianobakteria telah dicirikan dengan kaedah metagenomiks, pencilan sianobakteria termofilik masih belum dilakukan dan dicirikan secara mendalam. Berbanding dengan sianobakteria mesofilik, maklumat mengenai ciri-ciri penggalak tumbesaran pokok (PGP) masih belum dikaji. Memandangkan jumlah tanah pertanian semakin kurang untuk aktiviti bioprospek mikrob, sumber alternatif untuk pencilan mikrob yang mempunyai ciri-ciri PGP amatlah diperlukan untuk tujuan penghasilan baja bio. Jadi, kajian ini dijalankan untuk tujuan pemencilan dan pencirian sianobakteria dari kolam air panas tempatan – Sungai Klah (SK) dan Lubuk Timah (LT) di negeri Perak dengan menggunakan kaedah pencirian morfologi dan molikular. Penghasilan hormon tumbesaran, IAA daripada kultur axenic telah diukur. Ciri-ciri PGP juga dinilai dengan rawatan benih padi dalam ekstrak air sianobakteria. Sejumlah enam strain bakteria telah dipencilkan daripada kedua-dua kolam air panas. Strain LTM dan LTW dikenalpasti sebagai Leptolygnbya sp. manakala strain SEM, SEH, STH and STM dikenalpasti sebagai *Thermosynechococcus elongatus*. Kesemua enam strain menghasilkan kepekatan IAA dalam linkungan 670.10 pg/μL ke 2010 pg/μL. Ekstrak air juga didapati meningkatkan aktiviti enzim amilase dalam benih padi dari 5 hari selepas percambahan (DAG) ke 10 DAG. Secara amnya, penghasilan IAA dan peningkatan aktiviti amilase dalam benih yang dirawat dengan ekstrak air berkemungkinan membantu untuk menambahbaik panjang akar, pucuk dan nisbah akar kepada pucuk (RS). Kesimpulannya, sianobakteria termofilik daripada kolam air panas boleh dieksploitasikan sebagai sumber baru mikrob yang mempunyai ciriciri PGP untuk penghasilan baja bio pada masa depan.

**Highlights**

- A total of six thermophilic cyanobacteria strains were isolated from Sungai Klah (strains SEM, SEH, STH and STM) and Lubuk Timah (strains) LTM and LTW) hot springs located in the Perak state of Peninsular Malaysia.
- All six strains produced a plant growth promoting hormone, indole acetic acid (IAA) at concentrations ranged from 670.10 pg/μL to 2010 pg/μL under *in-vitro* condition.
- Rice seeds primed with cyanobacterial water extracts have shown an overall increase in the longest root length, shoot length and root-to-shoot (RS) ratio of rice seedlings.

## INTRODUCTION

Cyanobacteria are a large and diverse group of prokaryotes that can be found thriving in most Earth ecosystems ranging from freshwater rivers and lakes, seas to extreme environments such as deserts, hot springs and the polar regions (Radzi *et al*. 2019). As the only bacteria that carry out photosynthesis, the cyanobacteria are of great interest to researchers due to several fascinating characteristics. Besides being able to grow as extremophiles, they play key roles in the global nitrogen and carbon cycles. They are also widely studied for their potential in biofuel production as well as a myriad of biotechnological applications (Lau *et al*. 2015; Noreña-Caro & Benton 2018). Due to their metabolic plasticity, cyanobacteria are also known to produce metabolites with promising therapeutic properties (Brito *et al*. 2015; Nandagopal *et al*. 2021).

Malaysia is home to numerous hot springs where the water arises from groundwater sources is heated geothermally as compared to volcanic hot springs located in Yellowstone National Park (United States), Japan, India, New Zealand, Indonesia and the Philippines (Papke *et al*. 2003; Chan *et al*. 2015; Prihantini *et al*. 2016; Masaki *et al*. 2016; Martinez-Goss *et al*. 2019). The presence of cyanobacterial community in hot springs of Peninsular Malaysia has been discovered using the culture independent *16s rRNA* metagenome sequencing approach (Chan *et al*. 2015; Chan *et al*. 2017; Lee *et al*. 2017; Halim *et al*. 2020). Nonetheless, the diversity of cyanobacteria in these hot springs remains unclear since most of these studies emphasised on the elucidation of the thermophilic microbial community in general. It was briefly mentioned that Sungai Klah hot spring from Sungkai, Perak, Malaysia comprised of cyanobacteria from the genera of *Cyanobacterium*, *Synechococcus*, *Gloeobacter* and *Oscillatoria* (Chan *et al*. 2015). In addition, the morphological and molecular features of culturable cyanobacteria isolates from the Malaysian hot springs are not well-characterised. There is only one study that described different culturable strains of thermophilic *Synechococcus elongatus* from Kedah, Perak, Selangor and Negeri Sembilan can be clustered into two major groups based on intracellular phycocyanin concentration (Siva & Salleh 1997).

Bioprospecting for thermophilic plant growth promoting (PGP) bacteria for agricultural applications has received much attention recently due to their ability to sustain plant growth under adverse environmental condition (Rodriguez & Durán 2020). Thermophilic bacteria from the genus *Bacillus* isolated from agricultural soils were found to improve wheat growth (Santana *et al*. 2013; Santana *et al*. 2020). Tomatoes inoculated with a consortium of thermophilic *Bacillus* species derived from a native desert flower have also shown improved growth when grown at different levels of water limitation (Astorga-Eló *et al*. 2021). Similarly, a consortium of thermophilic *Bacillus* species from hot springs enhanced the field production of mung bean when cultivated under drought condition (Verma *et al*. 2018). Although the PGP properties of thermophilic cyanobacteria is not known, their mesophilic counterparts have proven to improve agriculture crops through the production of essential growth-promoting regulators, vitamins, amino acids and the solubilisation soil mineral nutrients for efficient plant uptake (Rai *et al*. 2019; Múnera-Porras *et al*. 2020; Toribio *et al*. 2020). Moreover, due to extensive agricultural activities to feed the overpopulated world, the amount of arable land has dwindled which has also accelerated the loss of microbial diversity (Wolińska *et al*. 2017). Consequently, bioprospecting for microbes with beneficial traits becomes more challenging than before. This heightens the need to search for alternative natural source for bioprospecting microbes with PGP properties.

In this study, cyanobacteria isolates were isolated, purified and identified from two local hot springs located in the Perak state of Malaysia through morphological and molecular characterisation. The purified isolates evaluated for their PGP properties in improving the growth of rice seedlings using the seed priming method.

## MATERIALS AND METHODS

### Sampling Sites

**Figure 1:**
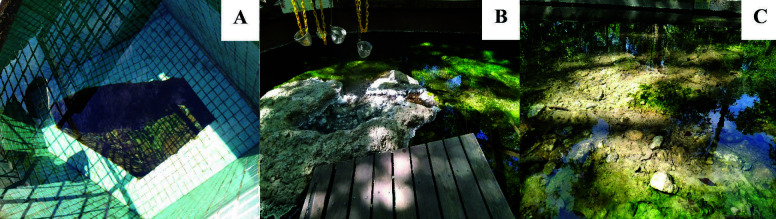
Hot spring water and cyanobacteria mat sampling locations of (A) main hot spring source of Lubuk Timah hot spring, (B) egg boiling area, and (C) therapeutic area of Sungai Klah hot spring.

Hot spring water samples and cyanobacterial mats were collected from two hot springs in Perak, namely Sungai Klah (SK) hot springs, Sungkai (N3°59′53.262″ E101°23′29.757″) and Lubuk Timah (LT) hot springs, Ipoh (N4°33′29.928″ E101°9′54.434″). Mat sample collection was performed by scraping the rock surfaces with a sterile spatula and stored temporarily in a 1 L vacuum flask filled with hot spring water. Water sample was also collected into a vacuum flask. The samples were transported immediately back to lab for culturing. The sampling locations of SK and LT hot springs were depicted in Fig. 1.

### Isolation and Purification of Cyanobacteria

Two isolation strategies were adopted in this study. Firstly, about 100 mg of cyanobacterial mat was homogenised in a sterile mortar and pestle before it was serially diluted using BG-11 broth. The diluted samples were spread onto BG-11 agar. Similarly, the water samples were serially diluted in BG-11 broth and spread onto BG-11 agar. Secondly, about 100 mg of cyanobacteria mat or 5 mL of water samples were transferred to 200 mL of BG-11 broth to enrich the cyanobacteria population. After growth was observed, the enriched culture was serially diluted and spread on BG-11 agar. The samples were incubated at 50°C under the illumination of cool-white, fluorescent lamps (~3,000 lux) for 21 to 30 days until growth was observed. Culture purification was performed through several passages of streak plating until axenic cultures were obtained.

### Morphological Characterisation

The morphological features of the axenic cyanobacteria cultures were observed under a compound microscope (Motic microscope BA310, USA) equipped with a camera (Moticam A16, USA). The morphology and cellular size of the isolates was captured and measured using Motic Images Plus 2.0 ML software (USA). The morphological features of these isolates were characterised and the descriptions were matched with morphological references from Desikachary (1959), Castenholz (2001), Komárek (2007), Katoh *et al*. (2001), Komárek and Johansen (2015), and Komárek *et al*. (2020).

### Genomic DNA Isolation

Genomic DNA was isolated according to Tillet and Neilan (2000) with slight modifications. Four-week-old cyanobacteria cells were harvested at 12,000 rpm at room temperature for 10 min. The cells were suspended in 50 μL TE buffer and homogenised using sterile mortar and pestle. The slurry was then added with 750 μL of XS buffer (1% [w/v] potassium ethyl xanthogenate, 100 mM Tris-HCl pH 7.4, 20 mM EDTA pH8.0, 800 mM ammonium acetate and 1% [w/v] SDS). The mixture was incubated at 70°C for 2 h followed by a 30 s vortex and 30 min of incubation on ice. The sample was centrifuged at 12,000 rpm for 10 min and the supernatant was added with 0.7 volume of ice-cold isopropanol. The sample was incubated at room temperature for 10 min and centrifuged at 12,000 rpm for 10 min. The supernatant was discarded and the DNA pellet was washed twice with 70% (v/v) of ethanol. The pellet was air dried in a laminar air flow for 15 min and resuspended in sterile distilled water. The DNA samples were stored in −20°C until further use.

### PCR Amplification and Sequencing

Two cyanobacteria-specific primer sets, thereby named as primer N and G, derived from Nübel *et al*. (1997) and Genuário *et al*. (2013) were used respectively in this study (Table 1). Both primer N and G amplify the 16s rRNA and 16–23s rRNA region, respectively. The PCR amplification was performed in a Bio-Rad T-100 Thermal Cycler (Bio-Rad, USA) using 25 μL reactions containing 10 ng of genomic DNA, 0.4 μM of each oligonucleotide primers, 0.2 mM of each dNTP, 2.0 mM MgCl, l PCR buffer (20 mM Tris-HCl (pH 8.4), 50 mM KCl), and 1.5 U GoTaq^®^ DNA polymerase (Promega, USA). The thermal cycling conditions for each primer sets used in this study were as described in Nübel *et al*. (1997) and Taton *et al*. (2003), respectively. PCR products were separated on a 1% (w/v) agarose gel (1 × TBE buffer) by electrophoresis at 80 V for 40 min and visualised with a Bio-Rad ChemiDoc™ Imaging System equipped with Image Lab software (Bio-Rad, USA). The PCR products were then sent for Sanger sequencing at Apical Scientific Sdn. Bhd. (Malaysia).

### Molecular Characterisation

The nucleotide sequences obtained were manually edited to address any insertions, deletions of nucleotides and to remove any noisy sequences. Multiple sequence alignment of the sequences from this study and reference sequences from the GenBank NCBI database was conducted using the clustalW algorithm to generate a phylogenetic tree using the neighbour-joining method. Distance matrices among aligned sequences were calculated using the Kimura two-parameter method. A bootstrap analysis of 1,000 replications was used to estimate the robustness of the phylogenetic tree. All these operations were performed using the MEGA11 software (Tamura *et al*. 2021). The gene sequences for the cyanobacterial isolates were also submitted and deposited at the GenBank NCBI database.

### Determination of Indole Acetic Acid (IAA) Production

A loopful of cyanobacteria was cultured onto BG-11 broth supplemented with 150 mg/L of tryptophan and incubated for 30 days. A volume of 1 mL of the broth was added to 2 mL of Salkowski reagent. The mixture was incubated for 30 min in dark and absorbance was read at 540 nm. A standard curve was constructed using IAA and result was expressed as pg/mL of IAA produced.

### Preparation of Water Extract

The axenic culture of cyanobacteria was grown for 30 days in 100 mL of BG-11 broth in the same condition as described above. The water extract of cyanobacteria was prepared according to Buono *et al*. (2021) with slight modifications. A total of 1 g of fresh mat was homogenised in a sterile mortar and pestle before adding 100 mL of sterile distilled water to make 1% (w/v) of water extract. The extract was left incubated on an orbital shaker at 150 rpm, room temperature for 24 h. The extract was then stored at −20°C until further use.

### Rice Seed Germination and Growth Assay

The MR 297 rice seeds were obtained from an authorised local seed producer, Kilang Beras Seri Merbok Sdn. Bhd., Kedah, Malaysia. The seeds were surface sterilised in 20% (v/v) Clorox solution for 20 min and rinsed several times in sterile distilled water. A total of 30 rice seeds was imbibed in the cyanobacterial water extract at room temperature for 24 h. The seeds were then transferred onto a petri dish overlaid with moistened tissue paper. The seed germination rate, shoot length, longest root length and root-to-shoot (RS) ratio were measured after 7 days of germination (DAG). The experiment was repeated twice.

### Alpha Amylase Activity Assay

The rice seed amylase assay was conducted according to Afiukwa *et al*. (2009) with slight modifications. The rice seeds were surface sterilised and germinated as described above. The amylase activity was measured at day 5, 7 and 10 DAG. The shoot and root were removed from the germinating seeds. A total of 10 seeds was grounded on ice using a mortar and pestle. A volume of 10 mL of cold distilled water was added to the homogenate and mixed well. The homogenate was then transferred to a 50 mL centrifuge tube and added to 20 mL of final volume using cold distilled water. The samples were incubated on ice for 10 min with occasional shaking. Subsequently, the extract was spun at 6,000 rpm for 10 min at 4°C. One milliliter (1 mL) of the crude enzyme extract was added to 1% (w/v) of soluble potato starch which was previously prepared in 0.1 M sodium acetate buffer (pH 4.8). The samples were incubated for 15 min at 40°C. To terminate the enzymatic reaction, 3 mL of 10% (v/v) of HCl was added to the samples followed the addition of 100 μL of Lugol’s iodine. Absorbance was read at 620 nm and the result was expressed rate of starch hydrolysis (μg/mL) per min.

### Spearman Correlation Coefficient

The non-parametric test method of the Spearman correlation coefficient was used in this study to measure the relationship between two variables. In this study, the variables such as the amount of IAA produced, growth of rice seedlings and the rate of starch hydrolysis were subjected to correlation analysis to investigate the relationship between these variables. The correlation degree was based on the grading standards of the coefficient values, ρ as shown in Table 2 (Yan *et al*. 2019).

### Statistical Analysis

The data obtained from each experiment was analysed by one-way ANOVA in a completely randomised design. Mean values were compared by Duncan’s multiple range test at *p* ≤ 0.05 significance level by using the SPSS software version 21.0 (IBM, USA).

## RESULTS

### Morphological Features Cyanobacteria Isolates

**Figure 2:**
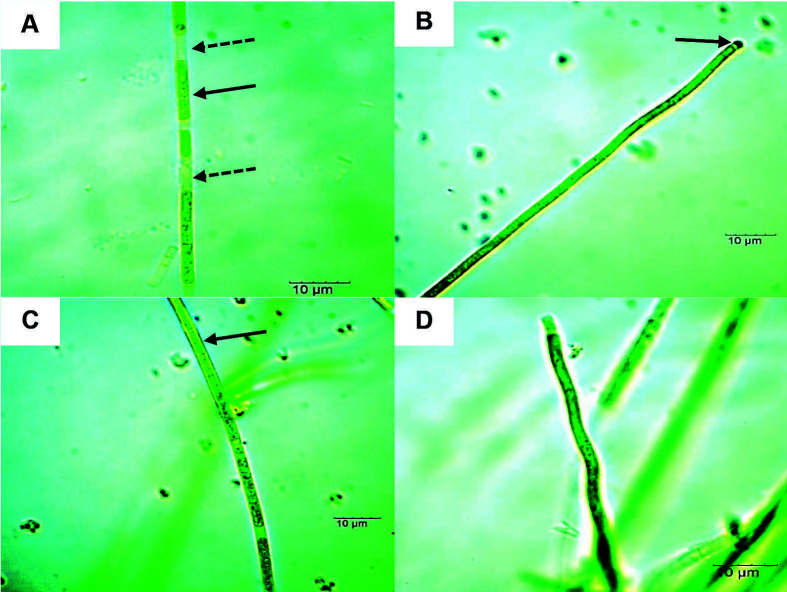
Light micrographs of LTM strain (*Leptolyngbya* sp.) under 100× magnification. Notable morphological structures were depicted as: (A) Hormogonia (indicated by arrow); necridic cells (indicated by dotted arrow), (B) straight trichome with rounded apical cells (indicated by arrow), (C) sheath encapsulated trichome (indicated by arrow) and (D) curved to bent trichome. Bars = 10 μM.

**Figure 3:**
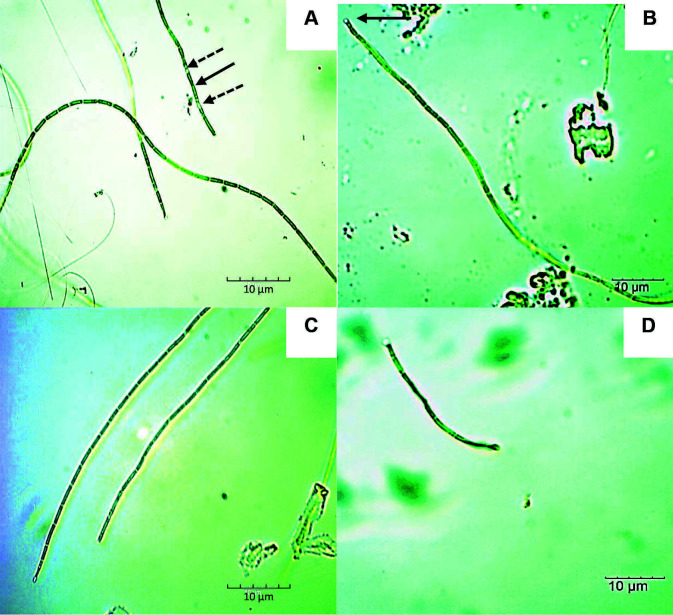
Light micrographs of LTW strain (*Leptolyngbya* sp.) under 100× magnification. Notable morphological structures were depicted as: (A) Hormogonia (indicated by arrow); necridic cells (indicated by dotted arrow); curved trichome arrangement, (B) straight to bent trichome with rounded apical cells (indicated by arrow), (C) sheath encapsulated trichome and (D) fragmented trichome. Bars = 10 μM.

A total of six isolates of filamentous cyanobacterial strains from the Oscillatoriales order were obtained from SK and LT hot springs. Two isolates from LT hot springs (strain LTM and LTW) were identified morphologically as *Leptolyngbya* sp. Both isolates reproduced by forming hormogonia which were separated by necridic cells (Figs. 2A and 3A). The hormogonia was then separated into individual filaments (Fig. 3D). The trichomes were uniseriate and arranged as straight, bent to curved filaments (Figs. 2A, 2D, 3A, 3B and 3C). The trichomes are encapsulated in thin sheaths (Figs. 2C and 3C). The apical cells are slightly attenuated and rounded (Figs. 2B and 3B). The major morphological difference between these two strains was the cell morphology. The LTM strain has short cylindrical, isodiametric cells compared to LTW strain which has elongated, cylindrical cells. Both strains did not exhibit any true or false branching.

**Figure 4:**
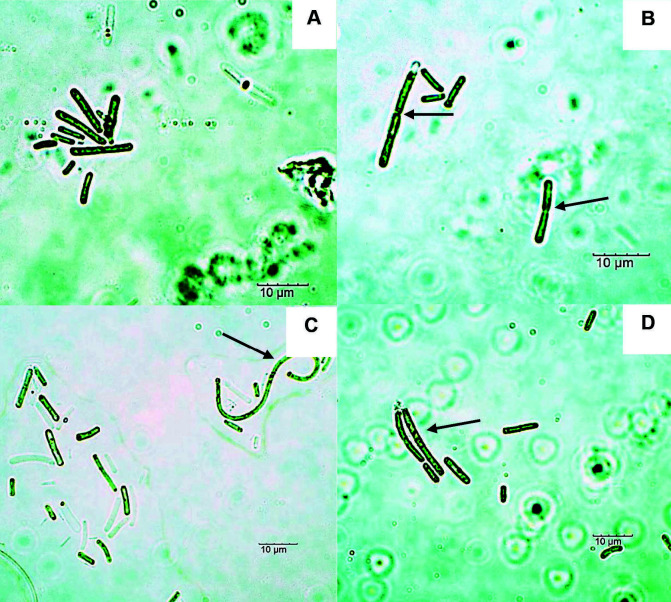
Light micrographs of SEM, SEH, STH, STM strains (*T. elongatus*) under 100× magnification. Notable morphological structures were depicted as: (A) solitary rod-shaped cells, (B) cell division by binary fission (indicated by arrow), (C) polar granules (indicated by dotted arrow); cell filamentation (indicated by arrow) and (D) cell filamentation (indicated by arrow) as observed in strain SEH and STM. Bars = 10 μM.

A total of four isolates was obtained from SK hot springs (Table 3). Strains SEM, SEH, STH and STM were morphologically identified as *Thermosynechococcus elongatus*. They are characterised by their solitary rod-shaped cells which can be straight to slightly curved and the trichomes lacked structured sheath (Fig. 4A). Only strain SEH and STM showed the formation of cell filamentation (Figs. 4C and 4D). These isolates reproduced through binary fission (Fig. 4B). The cells were cylindrical with granular content and rounded apical cells.

### Phylogenetic Analysis of Cyanobacteria Isolates

**Figure 5:**
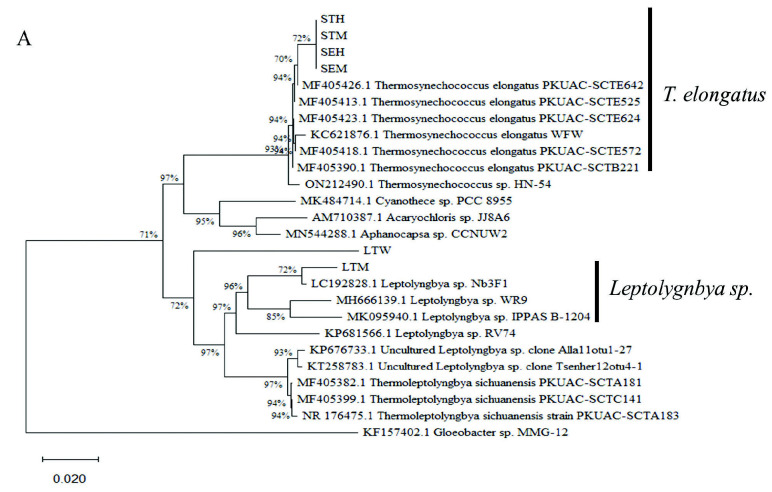

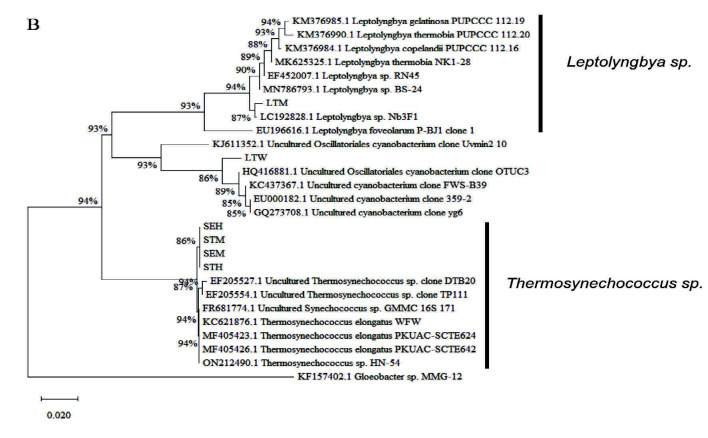
Neighbour-joining trees inferred from (A) Primer G 16s–23s rRNA (~1,400 bp) and (B) Primer N 16s rRNA (~400 bp) gene sequences. The values at the nodes indicate bootstrap values with 1000 replicates. The sequences determined in the present study are indicated in abbreviations (LTM, LTW, SEM, SEH, STH and STM). The *Gloeobacter* sp. MMG-12 sequence was designated as an outgroup. The scale marker represents 0.02 nucleotide substitution per sequence position.

The gene sequences derived from primer sets of Genuário *et al*. (2013) and Nübel *et al*. (1997) were deposited at NCBI-Genbank database in accession numbers of OQ102370 to OQ102381. Referring to phylogenetic tree in Fig. 5A, the sequences derived from primer G indicated that the LTM strain formed a clade with *Leptolyngbya* sp. strain Nb3F1 from Nakabusa hot spring from Japan, a saline strain WR9 from hypersaline salt marshes from an Indian desert, and a freshwater strain IPPAS B-1204 from the Tolbo Lake, Mongolia. Similarly, the LTM strain formed a clade with the same *Leptolyngbya* strain Nb3F1 when primer N was used for sequencing (Fig. 5B). Strain LTW formed a monophyletic clade with strains LTM and other *Leptolyngbya* strains with a high bootstrap value of 97% (Fig. 5A). On the contrary, strain LTW strain was separated from LTM and other *Leptolyngbya* strains in a paraphyletic branching of the phylogenetic tree inferred from primer N. Consequently, the LTW strain formed a clade with several unidentified and uncultured clones of cyanobacteria. All four SK strains (SEM, SEH, STH and STM) formed clade with *T. elongatus* strains PKUAC from hot springs located at Sichuan, China and a strain WFW from an unknown freshwater source from Taiwan (Fig. 5A). Phylogenetic analysis of the sequences derived from primer N showed that these four strains formed a clade with similar *T. elongatus* strains PKUAC and WFW and uncultured *Thermosynechococcus* sp. strains DTB20 and TP111 from hot springs in Tibet and *Thermosynechococcus* sp. strain from HN-54 from unknown source (Fig. 5B).

### IAA Production

**Figure 6:**
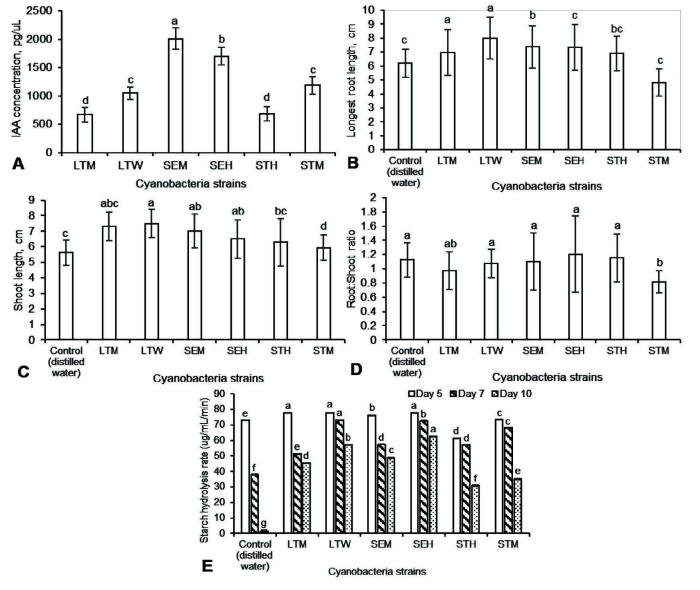
The production of (A) IAA of different thermophilic cyanobacteria strains from LT and SK hot springs and the effect of cyanobacteria water extracts on the (B) longest root length, (C) shoot length, (D) root-to-shoot ratio and (E) starch hydrolysis rate of germinated rice seedlings. Different letters indicate the mean values are significantly different (*p* ≤ 0.05).

The plant growth promoting (PGP) properties of the cyanobacteria isolates were evaluated. Strains SEM produced the highest concentration of IAA at 2,010.31 pg/μL, followed by strain SEH (1,701.03 pg/μL) (Fig. 6A). Both strains STM and LTW produced similar amounts of IAA at 1,185.57 pg/μL and 1,048.11 pg/μL, respectively. The lowest amount of IAA was produced for strains STH and LTM at 687.29 pg/μL and 670.10 pg/μL, respectively.

### Growth of Rice Seedlings

**Figure 7:**
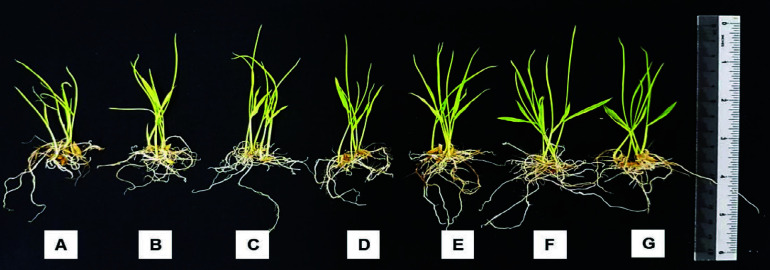
The overall growth of rice seedlings at 7 DAG after primed with (A) distilled water and water extracts of cyanobacteria strains (B) LTM, (C) LTW, (D) SEM, (E) SEH, (F) STH and (G) STM for 24 h.

The vegetative growth of rice seedlings was measured after imbibing the seeds in water extract of different cyanobacterial strains for 24 h. Overall, rice seedlings treated with strains LTW (7.98 cm), SEM (7.36 cm) and SHE (7.32 cm) exhibited longer root length compared to the control treatment (6.21 cm) (Fig. 6B). Strains LTM (6.98 cm) and STH (6.91 cm) did not produce root lengths that were statistically different when compared to the control treatment. In this study, only strain STM produced seedlings with the shortest root length (4.83 cm). On the other hand, strains LTM (7.3 cm), LTW (7.49 cm) and SEM (7.03 cm) produced longer shoot length as compared to the control treatment (5.62 cm) (Fig. 6C). There was no statistical difference among the rice root lengths measured for strains SEH (6.49 cm), STH (6.29 cm) and STM (5.94 cm). In terms of root to shoot ratio, there were generally no statistical differences among the cyanobacterial strains except for strain STM which had the lowest ratio (0.82) (Fig. 6D). Overall, the water extract of the cyanobacteria improved the vegetative parameters of the rice seedlings except for strain STM in comparison to the control treatment (Fig. 7).

### Seed Amylase Activity

The seed amylase activity reduced from 5 days after germination (DAG) to 10 DAG for all the treatments (Fig. 6E). In general, seeds that were imbibed with cyanobacterial water extract had overall higher starch hydrolysis rate compared to the control treatment. On the 5th DAG, high starch hydrolysis rate in rice seeds was observed in strains LTM (77.77 μg/mL/min), LTW (77.87 μg/mL/min) and SEH (77.78 μg/mL/min) whereas strain STH (34.97 μg/mL/min) showed the lowest hydrolysis rate compared to control treatment. On the 7th DAG, strains LTW indicated the highest hydrolysis rate (73.06 μg/mL/min), followed by strain SEH (72.54 μg/mL/min) and STM (68.35 μg/mL/min) while the control treatment showed the lowest rate (38.11 μg/mL/min) On the 10th DAG, strain SEH (62.69 μg/mL/min), LTW (57.15 μg/mL/min) and SEM (48.62 μg/mL/min) had higher hydrolysis rate compared to the other strains and control treatment (1.62 μg/mL/min).

### Correlation Analysis

A strong correlation (ρ = 0.627**) between the concentration of IAA produced and starch hydrolysis rate was observed (Table 4). The starch hydrolysis rate displayed a weak correlation between the longest root length (ρ = 0.317**) and RS ratio (ρ = 0.214*). The amount of IAA produced had a weak correlation between the longest root length (ρ = 0.265**) and RS ratio (ρ = 0.283*). The longest root length showed a strong correlation (ρ = 0.781**) with the RS values whereas the shoot length showed a negative correlation (ρ = –0.395**) with the RS values.

## DISCUSSION

In this study, a total of six cyanobacteria strains was isolated from LT and SK hot springs located in the Perak state. The major morphological feature that differentiated between the LTM and LTW strains were the cell size and width. The LTW strain had elongated, cylindrical cells compared to the short, cylindrical cells observed in strain LTM. Both strains did not show any true or false branching despite viewing the strains at different incubation period and culturing them at constant light and temperature. Komárek (2007) described that the elongated cellular phenotype is associated with *Protolygnbya* species, a subgenera of *Leptolyngbya*. Nonetheless, both LTM and LTW formed necridic cells which are crucial to the formation of hormogonia and separation from the trichome to form new filaments. *Protolygnbya*, on the other hand, does not form necridic cells (Komárek, 2007). In addition, both strains LTM and LTW fit the morphological features of the genus *Leptolyngbya* which is characterised by their thin trichomes (typically less than 3.5 μM in width) and the cell morphology can be isodiametric or have long cell lengths than widths (Komárek & Anagnostidis 2005; Bruno *et al*. 2009). Both primers N and G from Nübel *et al*. (1997) and Genuário *et al*. (2013) were used respectively for the molecular characterisation of strains LTM and LTW. The phylogenetic tree inferred from primers G and N indicated that strain LTM form a clade with other *Leptolyngbya* species. The LTW strain, however, formed a monophyletic clade with other *Leptolyngbya* species when primer G was used while it has a paraphyletic relationship with other *Leptolyngbya* species and formed a clade with unknown and uncultured cyanobacteria strains. As primer N covers only the 16s rRNA region, the use of primer G for molecular characterisation is a potential choice as it spans the 16s to 23s rRNA region which allows better resolution during phylogenetic analysis of cyanobacterial strains (Boyer *et al*. 2002; Martijn *et al*. 2019; Moore *et al*. 2019).

*Thermosynechococcus* was named based on its ability to grow in thermophilic temperatures compared to its mesophilic *Synechococcus* strains (Katoh *et al*. 2001). Four *T. elongatus* strains were isolated and purified from SK hot springs. Strains SEM, SHE, STH and STM were morphologically similar to the descriptions of Copeland (1936) and Katoh *et al*. (2001) with synonym as *T. vestitus* and basionym as *Synechococcus elongatus* var. *vestitus*. They form solitary short, cylindrical and filamentous-like cells (Katoh *et al*. 2001). Liao and Rust (2018) further explained that dim light condition induced filamentation in *Synechococcus elongatus* as a form of stress response during cell division. The molecular phylogeny analysis inferred from sequences derived from primers G and N revealed that all four strains in this study form a clade with other *T. elongatus* isolated from hot springs from China and Tibet.

Various studies have demonstrated the ability of mesophilic cyanobacteria strains to produce IAA which has been associated with improved plant growth but the thermophilic strains have not received attention (Kang *et al*. 2017). This is the first study to evaluate the PGP properties of thermophilic cyanobacteria from hot springs. All six strains produced IAA when grown in BG11-broth supplemented with tryptophan as the precursor. The exogenous IAA concentrations produced by these thermophilic strains were in the range of 670.10 pg/μL to 2010.31 pg/μL which were much lower than previously reported IAA concentrations (ranging from 10 μg/mL to 100 μg/mL) produced by mesophilic strains (Boopathi *et al*. 2013; Hussain *et al*. 2015; Duong *et al*. 2021). The low IAA produced could have led to the weak correlation between the longest root length and the RS values of the rice seedlings. Nevertheless, the overall growth of the rice seeds treated with the cyanobacteria water extracts was improved (except for strain STM) compared to seeds treated with distilled water only. Besides measuring the exogenous production of IAA, it is also crucial to measure the endogenous IAA extracted from the cyanobacteria (i.e., water extracts) to better correlate with the growth parameters of the seedlings. Various studies have reported the water extracts of mesophilic cyanobacteria contained cytokinin and auxin-like substances including IAA that have been applied to enhance plant growth and development (Singh 2014; Kollmen & Strieth 2022).

In cereal seeds such as rice, proteins and carbohydrates stored in the seed endosperm are readily mobilised during the germination process to provide sufficient energy and substrates for seedling development. During seed germination, the seed amylase activity is triggered to hydrolyse the stored starch to glucose (Liu *et al*. 2018). In this study, the starch hydrolysis rate of rice seeds treated with cyanobacterial water extract was measured in reference to the seed amylase activity. It was found that the starch hydrolysis rate is strongly correlated with the IAA concentration produced by the cyanobacteria strains. Low or high levels of IAA via direct application or microbial inoculation were found to significantly affect the amylase activity during seed germination and thus, affect the overall growth parameters of the seedlings (Ramakrishna & Rao 2006; Tabatabaei *et al*. 2016). From this study, the low levels of IAA produced by the thermophilic strains has, presumably, improved the starch hydrolysis rate of the seeds and contributed to the strong correlation between the longest root length and RS values. As seeds produced roots first during the early stages of germination, the activated amylase activity as triggered by the presence of IAA could have promoted root growth leading to higher RS ratio than the control treatment. The RS ratio is widely used as an indicator of plant health and its ability to compete for nutrient and water (Agathokleous *et al*. 2019). An overall higher RS ratio observed in rice seedlings treated with cyanobacteria water extracts (except for strain STM) may indicate a better capability in water and nutrient uptake compared to seedlings imbibed in distilled water only.

## CONCLUSION

Two *Leptolyngbya* sp. strains LTM and LTW were isolated and characterised from LT hot springs while four culturable *T. elongatus* strains SEM, SEH, STH and STM were obtained from SK hot springs. All six strains were morphologically identical to previously described species and molecularly related to homologous sequences from the GenBank. Rice seeds imbibed with water extracts of five thermophilic strains except for strain STM produced seedlings with improved vegetative growth compared to the control treatment. Future studies should also include characterising the PGP components in the water extracts to establish a better understanding the PGP effects on plant growth and yield. In other words, these potential strains could be further evaluated as potential seed priming agent in enhancing rice yield.

## Figures and Tables

**Table 1:** Primer sets used in this study.

Primer	Nucleotide sequence, 5′–3′	Targeted region	Reference	Expected size of PCR amplicon, bp
CYA359F	GGG GAA TYT TCC GCA ATG GG	16s rRNA	Nübel *et al*. (1997) Primer N	450
CYA781Ra	GAC TAC TGG GGT ATC TAA TCC CAT T			
CYA781Rb	GAC TAC AGG GGT ATC TAA TCC CTT T			
27F1 23S30R	AGA GTT TGA TCC TGG CTC AG	16s–23s rRNA	Genuário *et al*. (2013)	1,400
	CTT CGC CTC TGT GTG CCT AGG T		Primer G	

**Table 2:** Grading table of Spearman correlation coefficient (ρ).

Grading standards	Correlation degree
ρ = 0 no correlation 0 < |ρ| ≤ 0.19	Very weak
0.20 ≤ |ρ| ≤ 0.39	Weak
0.40 ≤ |ρ| ≤ 0.59	Moderate
0.60 ≤ |ρ| ≤ 0.79	Strong
0.80 ≤ |ρ| ≤ 1.00	Very strong
1.00	Monotonic relationship

**Table 3:** Spearman correlation coefficient (ρ) analysis between variables used in this study.

Variables	Longest root length	Shoot length	Root-to- shoot ratio	Starch hydrolysis rate	IAA concentration
Longest root length	1.000	0.182	0.781**	0.317**	0.265**
Shoot length	0.812	1.000	−0.395**	0.096	−0.120
Root-to-shoot ratio	0.781**	−0.395**	1.000	0.214*	0.283*
Starch hydrolysis rate	0.317**	0.096	0.214*	1.000	0.627**
IAA concentration	0.265**	−0.012	0.283**	0.627**	1.000

*Notes*:

* Correlation is significant at the 0.05 level (2-tailed);

** Correlation is significant at the 0.01 level (2-tailed)
